# Postoperative follow up in patients showing no evident residual disease - cut-offs for imaging/ intervention

**DOI:** 10.1186/1756-6614-6-S1-S5

**Published:** 2013-03-14

**Authors:** Michele N Minuto, Paolo Miccoli

**Affiliations:** 1 Department of Surgical Sciences, University of Genoa, IRCCS San Martino-IST, Largo R. Benzi 6, 16132 Genoa, Italy; 2 Department of Surgery, University of Pisa, Lungarno Pacinotti 43 - 56126 Pisa, Italy

## Abstract

The European Group generally agrees with the American guidelines on the issue of the indications for additional surgery in patients with recurrence of medullary thyroid cancer. The discussions have been focused mainly on the postoperative follow-up, where some European experts feel that a postoperative calcitonin-stimulating test is of some importance in assigning the patient to the “Cured” or “Non-cured” group immediately after surgery. A part of the European group feels that a negative calcitonin-stimulating test might lead to a less intensive follow-up in the late follow-up of these patients.

## Initial evaluation and treatment of postoperative patients

### Recommendation 73

*MTC serum tumor markers* (*Ct and CEA*) *should be measured 2–3 months postoperatively. Grade: B*

In the US guidelines it is stated that a stimulated Ct is not considered essential in the follow-up of the patient after surgery. The question that rose from the European panel was about its potential role in the postoperative course.

We should underline that some European experts believe that the role of stimulated Ct in the evaluation of complete success of surgery is essential for both the diagnosis and the follow-up of patients with medullary thyroid cancer. The stimulation can be performed with either pentagastrin (not easily available anymore also in Europe) or calcium (this test has been proved to be as effective as the pentagastrin test [1,2]).

When both the basal and the stimulated serum Ct are undetectable the patient is in complete biochemical remission and has about a 3% chance of biochemical recurrent disease during follow-up [3]. A negative postoperative stimulation test (defined by a Ct<10 pg/ml equal to <10 ng/L, as defined by Machens), is very restrictive and identifies patients that might undergo a less aggressive follow-up.

An European expert proposes a less restrictive cut-off of 30 pg/ml[ng/L]. The European board does not give any comment to this proposal.

### Recommendation 74

*When the postoperative basal serum Ct is undetectable* (*along with an undetectable stimulated serum Ct if performed*, *although the majority of the Task Force felt it was unnecessary*), *the risk of persistent or recurrent residual disease is low*, *and other tests or imaging techniques are not immediately required and the patient may enter into long-term follow-up. A neck US may be considered to establish a baseline. Grade: E Recommendation*

The European panel agrees on this recommendation. A short comment is made about the role of a postoperative “baseline” neck US in the case of a patient who is defined as “disease free” by serum Ct. Is it necessary to mention it in the guidelines, considering that it might be misinterpreted when performed by a non-expert radiologist?

## Testing and treatment of patients with detectable Ct

### Recommendation 75

*Postoperative MTC patients with detectable serum Ct levels* <*150 pg/mL should be evaluated with neck US. Grade: B Recommendation*

### Recommendation 76

*In addition to neck US*, *postoperative MTC patients with detectable serum Ct levels that are* <*150 pg/mL may be considered for additional imaging to serve as baseline examinations for future comparison even though these studies are usually negative. Alternatively*, *this additional imaging can be deferred and subsequently implemented should the serum Ct rise over time. Grade: C Recommendation*

It is unlikely to observe metastatic disease in patients with Ct < 150 pg/ml[ng/L]. The panelists agree with the American guidelines in defining the cut-off for further surgery only when there is morphologic evidence of metastatic disease, regardless of the Ct level. When surgery is indicated, the data available from the literature agree on the extreme rarity of a biochemical cure obtained after a second (or third) operation. Nevertheless, the reports also demonstrates that the overall prognosis might be improved in terms of survival.

### Recommendation 79

*In addition to neck US*, *postoperative MTC patients with serum Ct levels* ≥*150 pg/mL should undergo additional imaging techniques to evaluate for distant metastases. Grade: Recommendation B*

The European panel agrees on this recommendation without further comments.

## Initial evaluation and treatment of postoperative patients: role of invasive methods of staging

### Recommendation 86

*We do not recommend the routine use empiric liver or lung biopsies*, *hepatic vein sampling*, *systemic vascular sampling*, *or hepatic angiography prior to re-operation. These diagnostic procedures should be used sparingly*, *if at all. Grade: D Recommendation*

The European panel strongly agrees in the contraindication of these invasive and potentially morbid procedures.

## Patients with detectable serum Ct postoperatively, with negative imaging

### Recommendation 90

*Patients with detectable basal serum Ct levels postoperatively with negative imaging should have the basal Ct and CEA levels obtained approximately every 6 months initially to determine the DTs. Ongoing follow-up of these tumor markers and physical examination should occur at one fourth the shortest DT or annually*, *whichever is more frequent* (*i.e.*, *follow the patient every 6 months if the shortest DT is 24 months*)*. Grade: B Recommendation*

The European panel agrees on this recommendation without further comments.

### Recommendation 91

*In patients with detectable basal serum Ct levels postoperatively with negative imaging*, *if the Ct or CEA rises substantially since the previous anatomic imaging evaluation*, *then a neck US should be performed. The Ct elevation required to trigger this action typically depends on the basal serum Ct and the clinical situation*, *but elevation by more than 20% to 100% may prompt this evaluation. If the serum Ct is* >*150 pg/ml then systemic imaging should be repeated as well. Grade: C Recommendation*

A few European experts comment that citing cut-offs in these cases (last sentence) might be redundant, since imaging will always be indicated in such patients, regardless of any threshold.

## Long-term follow-up of patients in complete biochemical cure

### Recommendation 114

Long-term biochemical monitoring for patients with MTC who achieve a complete biochemical cure should be performed. Grade: B Recommendation

### Recommendation 115

Long-term biochemical monitoring for MTC patients who achieve a complete biochemical cure should include annual measurement of serum Ct. Grade: C Recommendation

The European experts take into consideration the possibility of a less regular follow-up after 10 years of “biochemical cure” of the disease, after the results described by Machens et al. [3] (JCEM 2005) who reported their incidence of recurrence in a range between 0.7-7.5 years. A new (and definitive?) stimulation test after 10 years is also proposed aiming at excluding the patients with a negative test from further tests.

## Patients with detectable serum Ct postoperatively, with negative imaging: long-term follow-up

### Recommendation 116

*Patients with persistent MTC should be monitored by measuring Ct and CEA levels*, *along with history and physical examinations. The timing of follow-up anatomic imaging may be based on the relative stability of these tests*, *presence or absence of symptoms*, *and the location of known or likely sites of metastatic deposits. Grade: C Recommendation*

### Recommendation 117

*Patients with detectable basal serum Ct levels postoperatively should have the basal Ct and CEA levels obtained approximately every 6 months to determine their DTs. Ongoing follow-up of these tumor markers and physical examination should occur at one fourth the shortest DT or annually*, *whichever is more frequent* (*i.e.*, *follow patient every 6 months if the shortest DT is 24 months*)*. Grade: C Recommendation*

### Recommendation 118

*In patients with detectable basal serum Ct levels postoperatively*, *if the Ct or CEA rises substantially since the previous anatomic imaging evaluation*, *then a neck US should be performed. The Ct elevation required to trigger this action typically depends on the basal serum Ct and the clinical situation*, *but elevation by more than 20–100% may prompt this evaluation. If the serum Ct is* >*150 pg*=*mL then systemic imaging should be repeated as well. Grade: C Recommendation*

See comments for Recommendations 90 and 91.

## Follow-up of patients without MTC at thyroidectomy

### Recommendation 119

*After prophylactic thyroidectomy demonstrates no evidence of MTC*, *the risk of developing MTC is low*, *and the optimal follow-up for these patients is uncertain. Annual measurement of basal serum Ct without measurement of CEA should be considered. Less frequent testing may be considered if there is no evidence of disease after prolonged follow-up. Grade: C Recommendation*

No further comments from the European panel.

## Role of stimulated Ct testing in the opinion of the American Experts

### Recommendation 120

*Stimulated serum Ct testing may detect low levels of residual disease despite undetectable basal Ct values. Such minimal disease is currently unlikely to be able to be localized or treated*, *and therefore this follow-up testing is not recommended* (*agreement amongst the Task Force was not unanimous*)*. Grade: D Recommendation*

The comment should underline, again, how differently the importance of the stimulation test is perceived by the Europeans; it is also important to notice how among the experts in the American panel there are also discordant opinions.

In European countries, stimulated Ct testing is commonly used both prior to and after the surgical treatment of medullary thyroid cancer, and is interpreted as a necessary tool by the majority of the clinicians involved in the management of the tumor.

## Management of CEA-positive but Ct-negative patients

### Recommendation 121

*Elevated CEA levels that are out of proportion to the serum Ct may occur from several causes*, *including some unrelated to MTC*, *which should be considered and evaluated as appropriate based on clinical judgment. Grade: C Recommendation*

No further comments are made from the European panelists.

## Conclusions

Postoperative follow up in patients showing no evident residual disease - cut-offs for imaging/ intervention

The European experts generally agree with the American guidelines for what concerns the indications and limits for additional interventions in patients who underwent surgery for medullary thyroid cancer.

The only different issue appears to be in the follow-up, and specifically in the role of a stimulating test that is felt important for the Europeans. A negative stimulation test might select those patients that might undergo a less aggressive follow-up because of their unlikely recurrence.

No other important issues have been discussed.

## List of abbreviations used

CEA: carcino embrional antigen; Ct: calcitonin; DT: doubling time; MTC: medullary thyroid carcinoma.

## Competing interests

No competing interest are declared by the two authors of this paper.

## Declarations

The funding for publication of this article came from the Department of Surgical Pathology, University of Pisa, Italy.

This article has been published as part of *Thyroid Research* Volume 6 Supplement 1, 2013: European comments on Medullary Thyroid Cancer Management Guidelines of the American Thyroid Association. The full contents of the supplement are available online at http://www.thyroidresearchjournal.com/supplements/6/S1. Publication of this supplement has been supported by the European Thyroid Association-Cancer Research Network.
